# Enhancing the In-Plane Behavior of a Hybrid Timber Frame–Mud and Stone Infill Wall Using PP Band Mesh on One Side

**DOI:** 10.3390/polym14040773

**Published:** 2022-02-16

**Authors:** Yinlan Shen, Xingchen Yan, Hui Liu, Guofang Wu, Wei He

**Affiliations:** 1 Key Laboratory of Urban Security and Disaster Engineering of Ministry of Education, Beijing University of Technology, Beijing 100124, China; shenyinlan@bjut.edu.cn (Y.S.); yanxingchenace@163.com (X.Y.); 2 Beijing Key Laboratory of Earthquake Engineering and Structural Retrofit, Beijing University of Technology, Beijing 100124, China; 3 Urban Renewal Research Office, Beijing Urban Construction Research Center, Beijing 101160, China; hui_liu1968@126.com; 4 Research Institute of Wood Industry, Chinese Academy of Forestry, Beijing 100091, China; 5 School of Civil and Transportation Engineering, Beijing University of Civil Engineering and Architecture, Beijing 100044, China; hewei@bucea.edu.cn

**Keywords:** traditional village dwellings, seismic reinforcement, polypropylene band meshes, timber frames, mud and stone infill

## Abstract

Traditional village dwellings in China consisting of timber frames with mud and stone infill walls represent an important part of cultural heritage and civilization. Due to the lack of an effective link between the wood frame and the infill and the poor cohesiveness of clay, the masonry infill can collapse during an earthquake, whereas the wood frame suffers minimal damage. In this study, current retrofitting techniques for village buildings were investigated and discussed. A method using polypropylene (PP) band mesh and cement mortar to retrofit the timber frame with a mud and stone infill was proposed and the connection construction details were designed. In-plane static cyclic tests were conducted on two full-scale wood–stone hybrid walls reinforced on one side with different grid sizes of the PP band mesh. The failure behaviors of the reinforced and non-reinforced sides of the specimens were compared, and the failure mechanics and seismic capacity of the two specimens, i.e., the strength, stiffness, ductility, and energy dissipation, were investigated. The results were also compared with those of a previous frame with stone infill without pebbles and no reinforcement. The study indicated that the retrofitting method strengthened the integrity and lateral resistance of the hybrid structure and prevented the collapse of the stone infill of the reinforced surface in a plane earthquake. The grid size of the PP band mesh substantially affected the lateral performance of the reinforced specimens. The hybrid wall with the narrow PP band mesh grid (150 mm × 150 mm) had a higher lateral stiffness (79%) and lateral capacity (50%) than the wall with the wide grid (250 mm × 250 mm). However, the narrow PP band mesh resulted in a lower ductility of the wall than the wide PP band mesh. The involvement of pebbles in the stone infill led to collapses sooner and a weaker lateral resistance than in the structure without pebble infill.

## 1. Introduction

**Figure 1:**
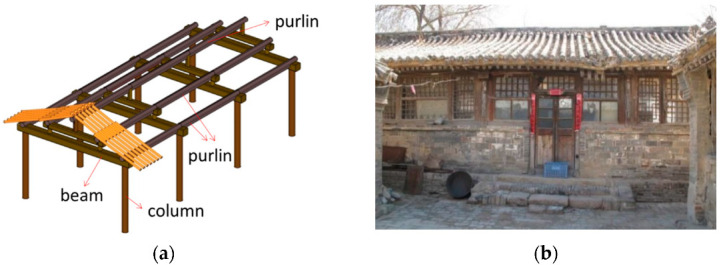
Traditional four-beam and eight-column village residence: (**a**) the four-beam and eight-column timber frame and (**b**) the timber frame with masonry infill wall.

Traditional Chinese village buildings are considered folk architecture. They are commonly found in rural locations and are constructed by local craftsmen using local raw material and traditional techniques with regional features. Traditional village dwellings represent a significant part of cultural heritage and civilization for humans worldwide. Traditional Chinese wood dwellings commonly built before the 1970s are the main type of traditional village architecture. The four-beam and eight-column-style residences represent typical old traditional dwellings in northern China, while traditional Chuandou style residences are more common in southern China. The Chuandou timber frames with infilled timber panels and numerous chuanfang, doufang, and dijiaofang are highly resilient in order to protect the inhabitants [1,2,3,4]. The four-beam and eight-column timber frames support most of the weight of the roof, with the remainder of the weight supported by the adobe, brick, and stone infill as enclosure and partition walls (Figure 1). Due to the lack of effective connection between the infill wall and the timber frame, the hybrid structure is prone to failure and the collapse of the infill wall, while the timber frame remains undamaged.

**Figure 2:**
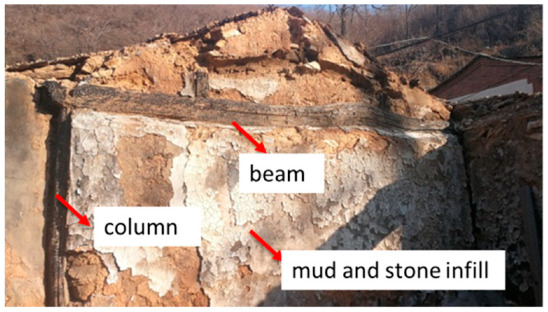
A timber frame with thick mud and stone infill.

Historically, wood frames with infill walls consisting of stone, mud, and brick were common in rural areas of countries all over the world, e.g., Portugal, Turkey, India, and Romania [5,6,7,8,9,10,11]. The typical construction practice is to use wood frames with diagonal bracing and cross connections because they ensure the deformation compatibility of the frame and the infill [12,13,14,15,16,17]. However, the four-beam and eight-column residences in China differ significantly from structures elsewhere in the world. The timber frame only relies on weak Mantou mortise-tenon joints without a horizontal tie beam or diagonal bracing. The weight of the gable roof is supported by eight wood columns resting on plinths or the ground without any fasteners, as shown in Figure 1a. A common practice is to use a thick infill wall between the timber columns to ensure the stability of the timber frame. Various rough stones of different sizes (e.g., stone blocks and rubble) are obtained locally and held together with clay mud by old, experienced masons to form a wall (Figure 2).

Traditional village residences with timber frame structures and infill walls in rural areas are decaying due to aging or cumulative seismic-induced damage. Natural damage also poses a threat to the preservation and safeguarding of historical folk architecture, which represents a significant section of many countries’ cultural heritage. Moreover, few people have the experience or knowledge required to rebuild old traditional village residences. Currently, the government promotes the protection, inheritance, and development of traditional villages. The protection of these traditional houses has become a trend, and reinforcing and repairing these buildings are important goals. A study of the seismic reinforcement methods of traditional hybrid timber–stone residences facilitates the protection of the important historical heritage of old traditional village buildings.

In this study, current retrofitting techniques for masonry structures are firstly investigated and discussed. Then, we propose for the first time a polypropylene (PP) band mesh retrofitting method based on the techniques used in brick masonry buildings for timber frame buildings with mud and stone infill walls. The focus is placed on retrofitting only one side (indoor reinforcement) of the hybrid timber–stone walls in order to preserve the architectural style of the traditional village buildings. Subsequently, full size specimens are constructed using representative traditional building methods and static cyclic in plane loading tests are conducted on two full-size reinforced specimens to determine the seismic performance of the hybrid walls. The failure modes of the unreinforced and reinforced sides of the walls and the lateral failure mechanics of the reinforced hybrid timber–stone walls are revealed. The hysteresis curves, strength and stiffness degradation, and energy dissipation capacity of the specimens are analyzed. The reinforcement effect of the structure is evaluated, and the influence of the different sizes of the PP band mesh on the in-plane seismic performance of the hybrid structure is discussed. Subsequently, the experimental results are also compared with those of a previous frame with stone infill without pebbles and no reinforcement. Finally, a conclusion and reinforcement suggestion are given. The results provide a fundamental understanding of the effectiveness of externally bonded PP band mesh and mortar as strengthening materials for unreinforced wood frames with mud and stone infill walls subjected to in-plane cyclic loading. This study is intended to provide information on the repair and protection techniques for existing traditional village dwellings in northern China.

## 2. Current Retrofitting Techniques for Village Buildings

In recent decades, multiple strengthening and repair techniques and products have been developed specifically for unreinforced brick or block masonry structures, including, but not limited to, post-tensioned steel reinforcing bars or strands; near-surface mounted steel reinforcing strips or fiber-reinforced polymer (FRP) strips; surface-bonded reinforcement, such as steel mesh and a mortar layer, PP band mesh and a mortar layer, polymer mortar, and fiber-reinforced cement composite shotcrete.

(1) FRP retrofitting technique

In 1997, Ehsani [18] used epoxy-bonded glass fabric to strengthen damaged masonry buildings subjected to the 1994 Northridge earthquake. ElGawady et al. [19] found that the lateral strength and stiffness of a masonry wall was 1.4–5.9 times higher after retrofitting using a one-sided carbon fiber-reinforced polymer (CFRP) bonding method. Chagas et al. [20] reinforced a masonry wall with vertical glass fiber-reinforced polymer (GFRP) strips. Similar studies on the cyclic loading of brick walls strengthened with vertical, horizontal, or diagonal CFRP strips or basalt fiber-reinforced polymer (BFRP) strips were conducted by Fam et al. [21], Marcari et al. [22], and Zhou et al. [23,24]. Tumialan et al. reported that the most common failure mode of FRP is de-bonding [25]. The low fire resistance and high cost of these materials and the expertise required to install them may represent obstacles to the widespread use of this retrofitting method for village buildings.

(2) Steel strip retrofitting technique

Yu et al. [26] applied angle iron plates on both panel’s sides with bolts anchoring to strengthen the integrity of adobe brick wall and timber columns, which ensure adobe buildings in Kashi to meet the demand of 8 degree seismic fortification intensity. Jing et al. [27] proposed steel plates externally bonded to both edges of a brick infill wall and bars embedded in mortar joints to reinforce timber frame with brick infill structures. The maximum load of the strengthened structure was increased by 27.8% and 20.6% and the ductility was improved by 32.5% and 82.5%, respectively, compared to the unreinforced structure. Borri et al. [28] adopted stainless steel strips mechanically attached to the sides of both panels using metal anchor bolts in the diagonal direction to reinforce brick walls. The lateral load capacity was 113% higher for the brick work panel and 66.7% higher for the dressed stone panels than the unreinforced panels. Farooq et al. [29] investigated the seismic performance of unreinforced burnt clay masonry wall panels strengthened with externally anchored steel strip mesh and evaluated the effect of different parameters on the performance improvement. Darbhanzi et al. [30] used steel strips as vertical ties on both sides of unreinforced brick masonry walls to strengthen the walls’ strength and ductility. However, the steel strip retrofitting technique has some limitations in the mud and rubble walls of traditional residences due to the poor cohesiveness of clay, the collapse failure of stones when anchoring the panel sides, and the mismatch with the historical architectural style.

(3) Post-tensioned wire rope retrofitting technique

The post-tensioned wire rope retrofitting technique incorporates unbonded prestressed wire ropes into structures to provide a self-centering mechanism to minimize the repair or reconstruction costs after an earthquake. Wight et al. [31] provided a brief review of the design and details of the first post-tensioned concrete masonry house with mortarless blocks in New Zealand. The in-plane seismic response of post-tensioned concrete masonry walls with openings was investigated using a shaking table test by Wight et al. [32]. Ma et al. [33] demonstrated the effectiveness of the external prestressing technique using shaking table tests of 1:4 scaled four-story brick masonry houses (non-prestressed and prestressed types). Yang et al. [34] proposed prestressed inclined and vertical wire ropes for retrofitting unreinforced masonry walls. The external prestressing technique was also applied to the reinforcement of brick masonry village houses by Liu et al. [35,36]. However, the post-tensioned wire rope retrofitting technique may not be suitable for the mud and stone walls of traditional residences because the stone walls are prone to collapse due to the low cohesiveness of the clay mud and the high stress on the top of the wall.

(4) Fiber-reinforced cement composite retrofitting technique

Catherin et al. [37] investigated the load-carrying capacity and deformability of unreinforced masonry scaled walls with textile-reinforced mortar (TRM) and FRP subjected to cyclic out-of-plane bending. Arisoy et al. [38] conducted an experimental study of retrofitting masonry walls with polyvinyl alcohol (PVA) fiber-reinforced cement stucco. The shear strengths of the solid and high-strength brick walls were approximately 1.5 times and 2.5 times higher, respectively, than that of a regular brick wall. Lin et al. [39] used engineered cementitious composite (ECC) to reinforce clay brick masonry walls in New Zealand. Deng et al. also conducted experiments using ECC [40]. Zhou et al. [41] proposed ECC splint reinforcement of an infill wall and reinforcing bars to strengthen the bond between the infill wall and the timber frame for timber frame-infill wall houses. However, the fiber-reinforced cement composite retrofitting technique has some limitations for reinforcing village buildings due to the large number and types of buildings in economically challenged areas.

(5) Welded wire mesh and mortar retrofitting technique

Several recent studies have been carried out to strengthen a masonry structure using welded wire mesh (WWM). Kadam et al. [42] evaluated the efficacy of ferro-cement (WWM with micro-concrete/mortar) for enhancing the shear and ductility capacity of masonry using diagonal compression tests of brick masonry panels. The reinforcement results showed an increase in the shear strength of up to seven times and an increase in the ductility of up to 24 times, with a reinforcement ratio of 0.29% in both directions. Shermi et al. [43] conducted diagonal axial compression tests of unreinforced brick masonry panels strengthened by WWM with different spacings (25 mm, 38 mm, and 50 mm) and a 1:3 cement to sand mortar mix. Messali et al. [44] performed quasi-static cyclic loading tests of full-scale hollow masonry brick walls with poor cementitious mortar repaired or strengthened with a thin layer of high-performance mortar and reinforced with a light steel mesh. A significant enhancement in lateral resistance was achieved for spandrel-pier specimens. The squat walls had rigid bodies, and the coating was undamaged after the test. The steel wire mesh-reinforced cement mortar method has also been used for reinforcing brick masonry residential buildings in rural areas of China, and was evaluated using a shaking table test in [45]. Most of these studies were conducted by embedding the WWM in the masonry wall [46]. However, steel wire mesh is very difficult to install in existing buildings because it is inconvenient to cut the mesh at the site. The reinforcement cost is much higher than that of using PP band mesh [47].

(6) Polypropylene (PP) band mesh and plastering mortar retrofitting technique

PP band mesh and plastering mortar have been extensively researched for use in retrofitting because the material is low cost and is easy to apply. Mayorca et al. [48,49] proposed a reinforcement method using PP band mesh and plastering mortar. Sugijopranoto et al. [50] found that adding PP band mesh to mortar increased the maximum force and improved the ductility in flexural tests compared to mortar blocks without the PP band mesh. Sathiparan et al. [51] conducted diagonal compression tests and out-of-plane tests of small specimens of non-retrofitted and PP-band retrofitted brick masonry walls. It was found that the retrofitted specimens had 2.5 times higher strength and 50 times higher deformation than the non-retrofitted samples in diagonal compression tests. In the out-of-plane tests, the retrofitted specimens had seven times higher strength and 60 times higher deformation than the non-retrofitted samples. Tests to determine the effect of the PP band mesh on the shear capacity of a brick masonry structure were conducted by Sathiparn et al. [52]. Shaking table tests of brick masonry buildings and adobe houses reinforced by PP band mesh were performed by Sathiparn et al. [53,54,55]. Zhou et al. [56] carried out low cyclic loading tests of PP band-reinforced brick masonry walls. The lateral capacity, energy dissipation capacity, and displacement ductility of the PP band-reinforced wall were 38–48%, 22–47%, and 138–226% higher, respectively, than those of the non-reinforced wall. Banerjee et al. [57] compared the effect of PP band mesh and steel wire mesh on the performance of a brick masonry wall in a flexural loading test using the four-point loading method. It was found that the strengthening techniques substantially delayed the collapse of the structure and enhanced the flexural strength and ductility. Wall specimens strengthened on both faces by steel wire mesh withstood an ultimate load that was more than 1.6 times higher than that of the PP band-strengthened wall. However, the PP band-strengthened wall contributed more to the deflection and energy ductility than the wire mesh-strengthened wall.

We can conclude the following based on the above studies. Most reinforcement studies focused on plastering mortar and brick walls, whereas few studies considered mud and stone walls, and there is a lack of research information on hybrid timber–stone structures. In traditional four-beam and eight-column hybrid timber–stone village residences, an in-plane interaction occurs between the wood frame and stone infill during seismic action. The stone infill consists of irregular stones of different sizes held together by clay mud with low cohesiveness. During an earthquake, out-of-plane failure of the stone infill wall or collapse can occur, resulting in the loss of property and lives. However, current reinforcement techniques are not suitable for the unique hybrid structure of traditional village houses. Reinforcement techniques for traditional village houses must be simple, effective, and low cost. They should meet seismic requirements and preserve the architectural style of the buildings. Therefore, we propose a PP band mesh retrofitting method for the hybrid timber frame–mud and stone infill structure, based on the PP band mesh retrofitting method used in brick masonry buildings.

## 3. PP Band Mesh Retrofitting Method for Wood Frame Structures with Mud and Stone Infill Walls

**Figure 3:**
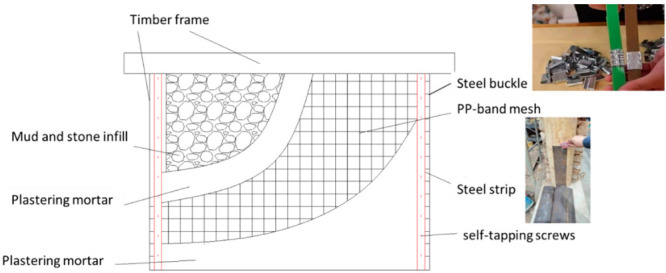
The proposed reinforcement method for the hybrid structure.

The original purpose of PP band mesh was for packing material. Here, the PP band mesh was attached to the stone wall, and plastering mortar was used to cover the surface of the mud and stone infill wall to strengthen its integrity. The edge of the PP band mesh was attached to the surface of the wooden columns using steel strips and self-tapping screws, as shown in Figure 3. Steel buckles were used to anchor the end of the PP band to keep it in place between the infill and the timber frame. The solid connection ensured good cooperation between the wood frame and stone infill. This reinforcement technique, as an affordable and simple strengthening strategy, requires less expertise from constructors and a minimum operating area, and uses raw materials that are available everywhere (PP band mesh, steel strips, steel buckles, etc.). In addition, the disturbance of the building’s residents is minimized during the strengthening, and the loss of usable space is minimal after strengthening.

## 4. Construction and Retrofitting Procedures

### 4.1. Timber Frame

**Figure 4:**
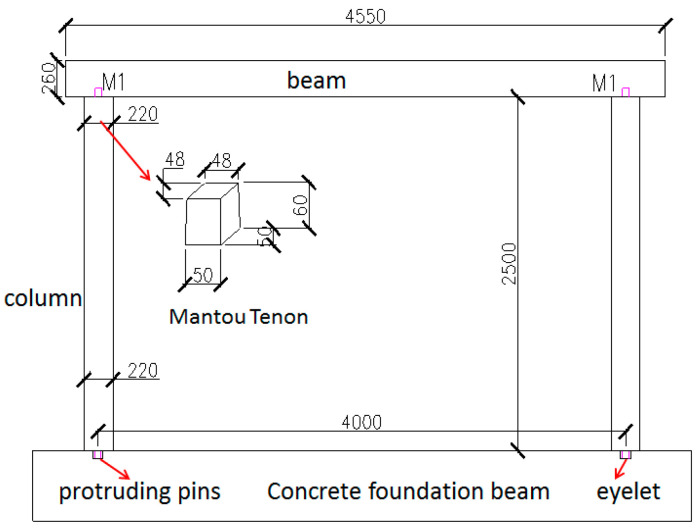
Details of the timber frame.

*Pinus sylvestris* grown in northern China was used for the timber frame in traditional village dwellings. The full-size timber frames were built by local carpenters who followed traditional construction practices according to a field investigation of representative timber dwellings. The horizontal beam had a round cross-section with a diameter of 260 mm and a length of 4.55 m. The columns had a spacing of 4 m, a diameter of 220 mm, and a height of 2.5 m, as illustrated in Figure 4. The connections between the beam and the column were weak Mantou mortise-tenon joints. The Mantou tenons with a 60 mm height had a cross-section of 50 mm × 50 mm at one end, and the cross-section decreased toward the top, where it was 48 mm × 48 mm. For a tight fit for the tenons, the mortises were also manually processed to be wider on the outside and narrower on the inside. In a traditional timber village dwelling, the timber columns are typically placed on corner stones, where the pins protruding from the column bottom are inserted into eyelets in the stone to allow movement at the base of the column. In the laboratory, concrete foundation beams with a cross-section of 0.5 m × 0.42 m and a length of 5 m were prefabricated and were used as the foundation. The protruding pins were fabricated at the bottom of the columns with a cross-section of 50 mm × 50 mm and a height of 60 mm. Two slots (80 mm × 80 mm × 60 mm) were cut in the top surface of each foundation beam at a spacing of 4 m to represent the eyelets.

### 4.2. Rubble Masonry Infill with Pebbles

**Figure 5:**
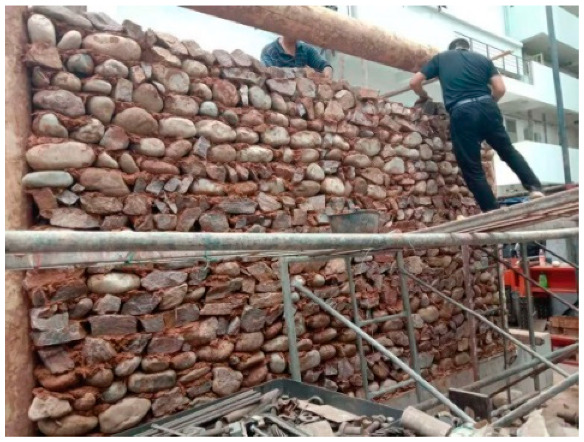
The masonry construction of the mud and stone infill wall.

The masonry construction of the mud and stone infill wall was conducted in the laboratory after the timber frames had been erected on the concrete foundation beam. Most of the raw construction materials used in the test, such as clay and stones, were obtained locally. The clay mud consisted of a mixture of clay, water, and 10–15 cm long straw. It was used as a mortar to layer the stones with diameter range of 20–30 cm. Local masons used traditional construction practices and placed a double layer of stones covered by a layer of clay mud, as illustrated in Figure 5. The infill walls had dimensions of 4 m long × 2.5 m high × 0.4 m thick, with the infilled pebble and stone each accounting for half.

### 4.3. Retrofitting Procedure

**Figure 6:**
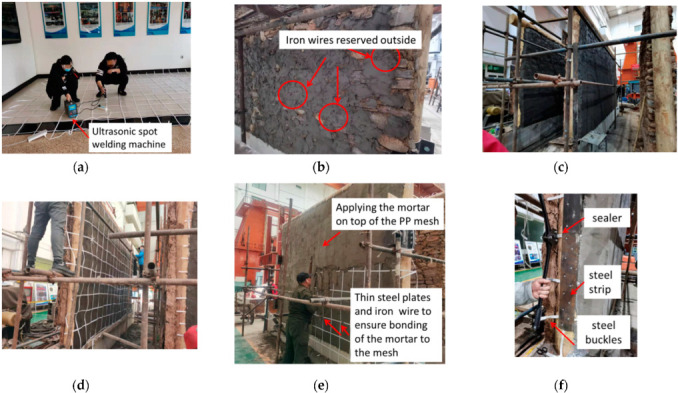
PP band retrofitting procedure for timber frame with mud and stone infill: (**a**) welding of the PP band mesh; (**b**) filling the gap using cement mortar; (**c**) applying the cement mortar at the bottom surface; (**d**) installing the PP band mesh; (**e**) applying the cement mortar at the top surface; and (**f**) anchoring the PP band connection between the timber frame and the infill.

After the clay mud of the infill had completely dried (more than 28 d), the PP band mesh and plastering mortar were used to strengthen the hybrid wall. The details of the reinforcement process are shown in Figure 6. The PP band mesh was fabricated using an ultrasonic spot welding machine (Figure 6a). Considering the number limitation of full-scale specimens and infilled stones with a diameter range of 20–30 cm in existing traditional village dwellings, two types of grid sizes were used for the PP band mesh—150 mm and 250 mm—in order to study the reinforcement effect of the structure and the influence of different sizes of the PP band mesh. Prior to applying plastering mortar, the wall surfaces were cleaned with water to remove loose material and to improve the adhesion between the mortar and stone masonry. The plastering mortar (cement, sand ratio = 1:4) was used to fill the gaps between the stones and obtain a flat wall surface. Then, iron wires curved manually into a U-shape were embedded in the mortar joints of the infill, and the ends were reserved outside for better fixing of the PP-band mesh to the wall surface (Figure 6b). The cement mortar was applied to the bottom surface of the wall (Figure 6c). Next, the PP band mesh was installed on top of the mortared surface of the wall (Figure 6d). The ends of the U-shaped iron wires were inserted in the grid holes to connect the mesh to the reinforced side. Thin steel plates (40 mm × 20 mm) were fixed on the top of the band mesh using iron wires reserved outside to ensure that the PP band mesh was embedded in the mortar and the mortar was attached to the mesh (Figure 6e). The mesh was wrapped around the column edge and was attached to the surface of the timber columns by the curved steel strip with self-tapping screws. The steel buckles were used to anchor the end of the PP band mesh using a sealer in order to prevent the removal of the connections between the infill and the timber columns (Figure 6f). Finally, the mortar was applied to the top of the wall surface. The overall thickness of the plastering mortar from the bottom of the flat surface to the top was about 20–30 mm. The specimen was watered for 7 d and remained under normal temperature conditions until the plastering mortar had sufficiently hardened.

## 5. Experimental Program

Since different types of stones are used for traditional village houses, two full-size timber frames with mud and stone infill consisting of pebbles and irregular stones were constructed in the laboratory. The same group of construction craftsmen who had performed the masonry work on the two walls using the traditional methods ensured the accuracy of the methods and the technical detail. One side of the hybrid walls was strengthened with PP band mesh and plastering mortar. As for the different grid sizes of PP mesh considered, specimen W1 had a mesh grid size of 250 mm × 250 mm and specimen W2 had a grid size of 150 mm × 150 mm (Table 1).

### 5.1. Mechanical Properties of Materials

**Figure 7:**
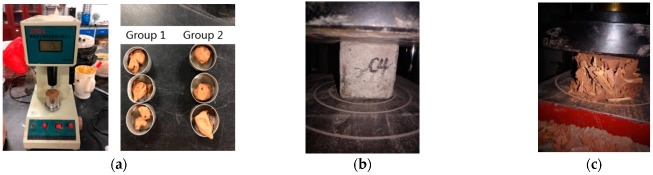
Masonry material tests: (**a**) liquid-plastic limit test of the clay; (**b**) compressive strength test of a block of plastering mortar; and (**c**) compressive strength test of a clay block.

Material tests of the plastering mortar and clay mud were conducted to obtain the mechanical properties of the masonry materials (Figure 7). The plasticity index of the clay (18.7) was obtained from a liquid-plastic limit combined test using a GYS-2 soil liquid-plastic limit combined measurement instrument (Nanjing Soil instrument Co., Ltd., Nanjing, China). The plastering mortar test samples had a size of 70.7 mm × 70.7 mm × 70.7 mm, according to the standard building mortar block compression test methods [58]. The average compressive strength of the six mortar cubes was 4.08 MPa, measured using an MTS material testing machine with a load range of 300 kN (MTS Systems Corporation, Eden Prairie, MN, USA). The average compressive strength of the clay cubes with the same dimensions of 70.7 mm × 70.7 mm × 70.7 mm was 1.73 MPa.

**Figure 8:**
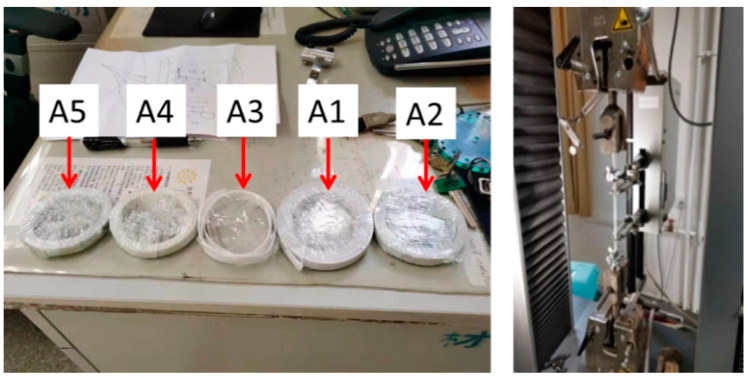
The five PP bands and the tensile test setup.

We selected five types of PP band products commonly available on the market for a tensile test (Figure 8) using the plastic tensile test standard [59] and a Zwick050 material testing machine with a load range of 50 kN (Zwick Roell group, Ulm, Germany). Table 2 summarizes the product dimensions of the PP band, the elastic modulus, and the ultimate tension capacity of the PP band specimens. Sample A3 with a width of 13 mm was selected as the reinforcing material for the hybrid timber–stone wall in the static test since it had the optimum ultimate tensile force and elastic modulus and was the widest PP band.

The mechanical properties of the wood (Pinus sylvestris, purchased from Beijing Zhihenghongchuang Trading Co., Ltd., Beijing, China) were also tested. The average elastic moduli of the wood in three directions (longitudinal (EL), radial (ER), and tangential (ET) directions) were 9420 MPa, 1039 MPa, and 520 MPa, respectively. The compressive strengths in the three directions were 28.6 MPa (EL), 6.1 MPa (ER), and 6.1 MPa (ET). The bending strength was 64.4 MPa.

### 5.2. Test Setup and Loading Protocols

**Figure 9:**
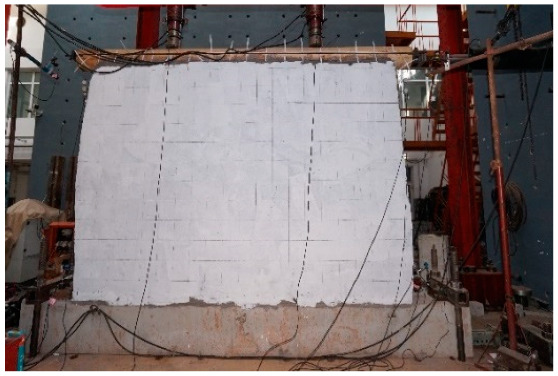
Specimen test.

The tests were conducted using a static cyclic loading system consisting of one horizontal hydraulic jack and two vertical hydraulic jacks. The horizontal hydraulic jack had a movement range of ±250 mm and the attached force sensor had a load range of 100 kN. The vertical hydraulic jack had a load range of 1000 kN and a horizontal movement range of ±150 mm. The vertical bearing load of the timber frame was about 38 kN, which was based on the roof’s weight and the snow load. A pre-compression load of 38 kN was applied at the top of the beam at one-third of the span. The horizontal load was applied by a horizontal hydraulic jack installed on the reaction wall. Two steel tie bars and a pin roll connecting the ends of the beam were used to move the hybrid wall in the horizontal direction. The vertical hydraulic jack was moved horizontally using rollers on a slide rail on the rigid beam. As shown in Figure 9, the concrete foundation beam was affixed to the laboratory ground by steel box beams and bolt anchors. The hydraulic jacks supported both ends of the foundation beam to prevent its slippage. During the test, manual control was applied to ensure a constant vertical load. Although the horizontal load was also manually controlled, the force sensor range of the testing system matched the lateral resistance of the specimens to ensure the reliability of the experimental data.

**Figure 10:**
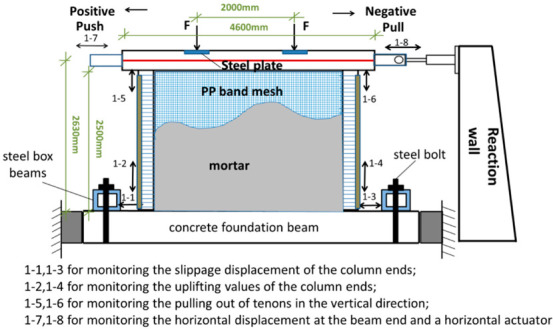
Test setup and measurement diagram.

In this test, eight displacement transducers were installed to monitor the deformation of different parts of the wall. The wire displacement transducers at the ends of the timber beam were used to monitor the lateral drift at the top of the wall. The displacement from the push of the horizontal actuator was defined as positive loading, and the displacement from the pull of the horizontal actuator was defined as negative loading. Dial gauges were placed at the top of the columns to measure the pulling out of tenons in the vertical direction and at the bottom of the columns to monitor the slippage and uplifting values of the column ends (Figure 10). However, no wire displacement transducers were used to monitor the rotation of the beam–column connections since they could be damaged by falling stones. The reinforced surface was coated with white emulsion paint, and a 200 mm × 200 mm grid was applied to observe the test phenomena.

**Figure 11:**
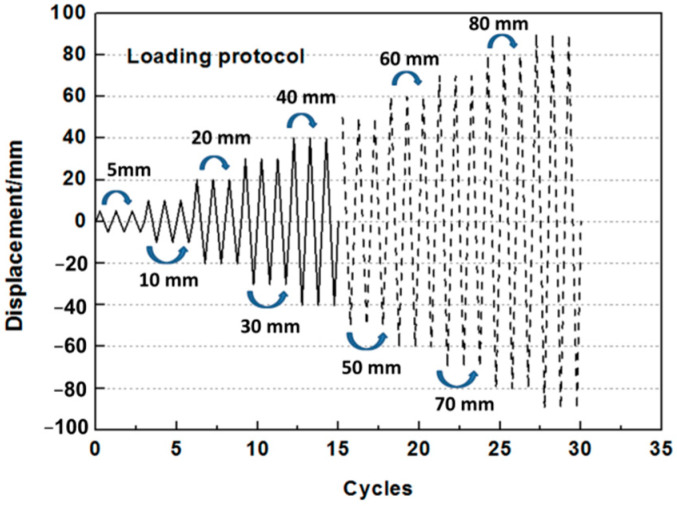
Schematic diagram of the loading protocol.

The static cyclic loading test used displacement control loading, and the loading speed was manually maintained at less than 2 mm/s; the loading protocols are illustrated in Figure 11. At a drift ratio of less than 0.5%, each level of horizontal loading displacement included three fully reversed cycles at equal amplitude, followed by an increment of 5 mm in the displacement loading level. Subsequently, each level included three fully reversed cycles of equal amplitude, followed by an increment value of 10 mm in the displacement loading level. The loading displacement increased up to the corresponding values according to the actual situation of the test. The displacement loading stopped when the monitored horizontal force of the walls was less than 80% of the peak lateral capacity during the test.

## 6. Result and Discussion

### 6.1. Experimental Phenomena of Strengthened Timber–Stone Hybrid Wall with PP Band Mesh

**Figure 12:**
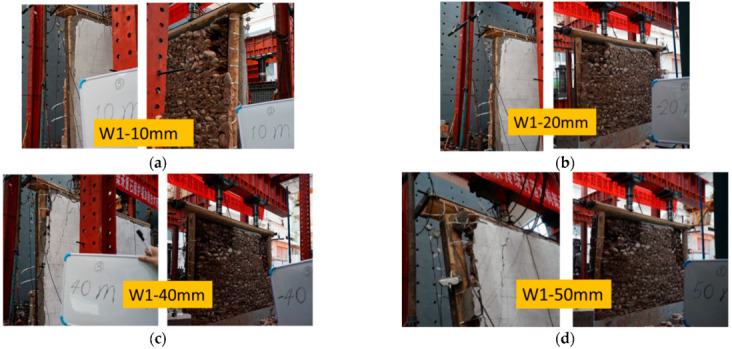
Displacement loading for W1: (**a**) 10 mm, (**b**) 20 mm, (**c**) 40 mm, and (**d**) 50 mm.

For specimen W1 with a PP band mesh grid of 250 mm × 250 mm, when the lateral displacement was within 5 mm, the wood frame and the infill operated in conjunction to resist lateral deformation. At 10 mm loading displacement, some stones fell at one corner of the unreinforced side, and a crack with a length equal to half the height of the wall was generated at the corner of the reinforced side, as shown in Figure 12a. The reason was the compression from the timber frame at the edge of the infill, causing stone dislocations in adjacent areas. After 20 mm loading displacement, the corner was crushed at the unreinforced side with a fan-shaped area of stones collapsed at both corners due to the diagonal bracing effect. In contrast, the reinforced side remained flat, and no stones fell out (Figure 12b). As the loading displacement increased to 40 mm, separation occurred between the timber frame and the infill, and greater gaps were observed at the upper end of the column and smaller gaps at the bottom. Meanwhile, the stones on the unreinforced side fell (about 1/8–1/6 of the wall area), whereas the reinforced side remained flat, except for a small amount of mortar falling from the upper end of the column (Figure 12c). When the loading displacement level reached 50 mm, the lateral force of the specimen dropped to less than 50% of the maximum capacity. The individual PP bands attached to the upper end of the column broke, and the reinforced surface remained flat, with some mortar falling from the upper end of the column (Figure 12d).

**Figure 13:**
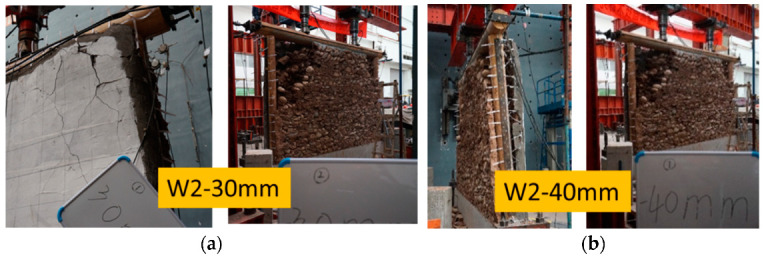
Displacement loading for W2: (**a**) 30 mm and (**b**) 40 mm.

For specimen W2 with a PP band mesh grid of 150 mm × 150 mm, at a loading displacement of 20 mm, a few stones fell at the corners of the unreinforced side and the mortar surface bulged, with multiple cracks appearing at the corners of the reinforced side, similar to W1. At a loading displacement of 30 mm, the reinforced side remained flat, except for a bulge and a deep crack at the corners. Due to the narrow grid size of the PP band mesh, a small amount of mortar fell from the upper end of the column. Meanwhile, a symmetrical collapse of the stones occurred at the corner of the unreinforced side, with a fan-shaped collapse area on the left (Figure 13a). In addition, the lateral force of specimen W2 dropped to below 50% of its maximum capacity. At a loading displacement of 40 mm, the compression from the frame caused detachment at the edge of the double-layer stone wall, and a collapse of the stones was observed on the unreinforced side (Figure 13b). The tension of the PP band mesh and the coating on the reinforced side prevented damage to the reinforced side. The individual PP bands attached to the upper end of the column exhibited tensile fractures.

**Figure 14:**
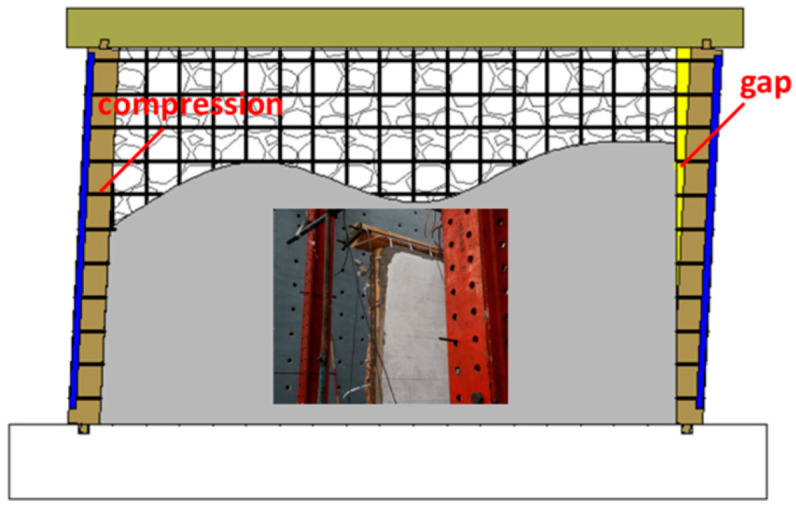
Deformation mechanism of the hybrid timber–stone wall strengthened by PP band mesh and cement mortar.

These observations indicate that the deformation incompatibility between the infill wall and the timber frame increases with an increase in the loading displacement. During low cyclic reversed loading, separation and squeezing occur alternately at the interaction surface between the infill wall and the frame. The diagonal bracing effect causes substantial extrusion pressure at the corner of the infill, resulting in falling stones. In addition, the compression of the frame causes the shear of the stone wall in the direction of thickness, leading to detachment at the edge of the double-layer stone wall. A bonding effect is observed between the cement mortar and the stones, especially at the corner of the infill. The PP band mesh attached to the timber frame prevents the out-of-plane stone collapse at the corner of the reinforcement surface due to the diagonal bracing effect, as shown in Figure 14. The reinforced side of the infill remains flat, with little damage and no falling stones. The PP band that connects the infill and timber frame also prevents the detachment of the reinforced side of the infill from the timber frame.

### 6.2. Hysteresis Curves

**Figure 15:**
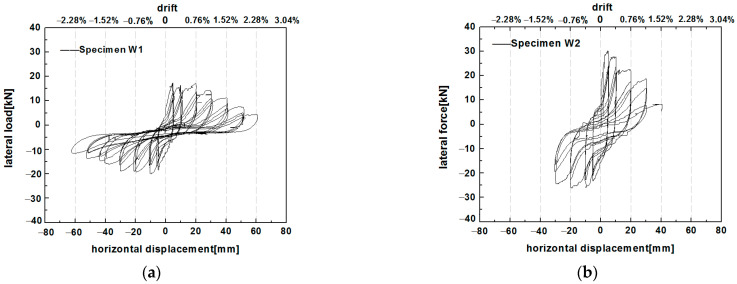
Hysteresis curves of (**a**) specimen W1 and (**b**) specimen W2.

The load-displacement hysteresis loops of the two specimens are illustrated in Figure 15. The lateral drift angle is the ratio between the top horizontal displacement and the height where the horizontal load is applied. The hysteresis loops of specimen W1 exhibited an 8-shape with plump ends and an extremely pinched waist, which indicates the internal slipping occurrence of the specimen. Before the loading displacement reached 10 mm, the hysteresis loops had a shuttle shape, indicating a high energy dissipation capacity. As the loading displacement increased, the infill cracked, and the stones started to slide. The hysteretic loops exhibited a reverse S-shape with a pinching of the curve. After the horizontal displacement reached 30 mm, the hysteresis curve approached a Z-shape, indicating substantial horizontal sliding due to the deformation incompatibility between the infill wall and the timber frame. The hysteresis curve of specimen W2 was shuttle-shaped with larger loops in early loading, demonstrating that the PP band mesh strengthened the wall and provided a high energy dissipation capacity. As the loading displacement reached greater than 20 mm, the hysteresis curve of specimen W2 approached an S-shape with a pinching effect.

Specimen W1 gained a strength of 17.24 kN at 5.14 mm in the positive loading direction, followed by a gradual decline in the lateral load. Meanwhile, a strength of 20.06 kN was obtained at a negative loading of 9.96 mm. Specimen W2 had a strength of 30.1 kN at 5 mm in the positive loading direction and a strength of 25.92 kN at 9 mm in the reverse loading direction. The hysteresis curves indicate that the grid size of the PP band mesh significantly affected the lateral strength and the fullness of the hysteresis curves. The lateral resistance was higher for W2 than for W1.

### 6.3. Idealized Equivalent Bilinear Curves

**Figure 16:**
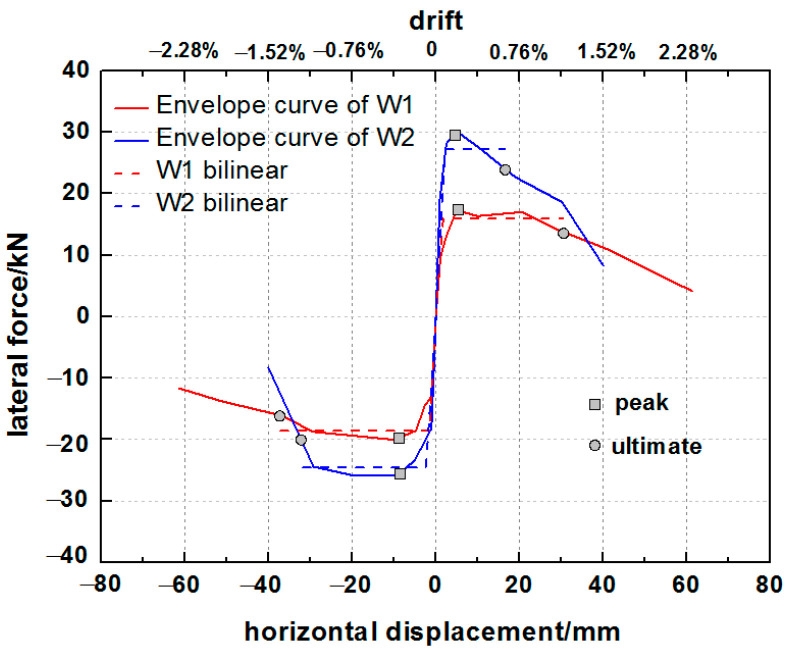
The envelope and bilinear curves of specimens W1 and W2.

The nonlinear hysteresis envelope curves of the two specimens strengthened with different grid sizes of PP band mesh are presented in Figure 16. The hysteresis envelope curves were idealized with bilinear elastic–plastic curves based on the equivalent energy elastic–plastic (EEEP) method using the ASTM 2126-09 standard [60]. The parameter values of the initial elastic stiffness (Ke), yield load (Pyield) and related displacement (Dyield), peak lateral load (Ppeak) and related displacement (Dpeak), failure load (Pu) and related displacement (Du), and ductility ratio (δ) are listed in Table 3.

Specimen W2 gained a higher initial stiffness (Ke; average of 18.98 kN/mm) than W1 (average of 10.61 kN/mm) with an increase of 79%, as well as a higher maximum lateral resistance (average value of 28.01 kN) than W1 (average of 18.65 kN), an increase of 50%. It should be noted that the grid size of the PP band mesh had a minimal effect on the displacement related to the strength limit state. The peak displacements were consistent for different grid sizes (about 5 mm for positive loading and 9–10 mm for negative loading). However, after the specimens reached the strength limit state, the hysteresis envelope curve of specimen W2 exhibited a faster decline than that of W1. Specimen W2 had a lower ductility value (10.35) than W1 (average of 20.75). The PP band mesh with a smaller grid resulted in higher stiffness and strength than the larger grid. Taking the ultimate displacement of specimen W1 as the division point (33.79 mm), the envelope curve of W2 before the division point was larger than that of W1. In addition, the reinforced side of the hybrid structure remained intact without collapsing or losing stones.

### 6.4. Stiffness Degradation Behavior

**Figure 17:**
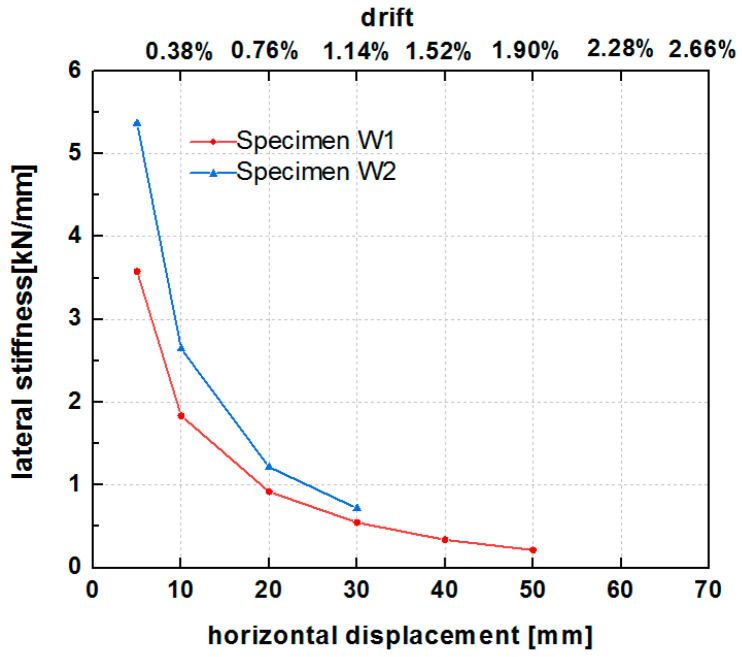
Stiffness degradation curves of specimens W1 and W2.

Based on the test standard [61], the cyclic secant stiffness was calculated for the cycle of each phase using the average slope of the line connecting the origin and the two points of the loads corresponding to the maximum displacement in the positive and negative loading directions. The stiffness degradation curves of the two specimens for increasing displacement and lateral drift are illustrated in Figure 17. The stiffness curves showed a rapid stiffness degradation in the early stage of lateral displacement loading, with the largest rate of decrease at lateral displacements of less than 20 mm (lateral drifts of less than 1%), followed by a gradual decline (20–30 mm). Subsequently, the lateral stiffness stabilized at greater than 30 mm displacement. The grid size of the PP band mesh affected the lateral stiffness of the hybrid structure. The stiffness of W2 was higher than that of W1 when the drift was lower than 1.14% (30 mm lateral displacement). At a horizontal displacement of 5 mm, the stiffness of W2 was 1.5 times that of W1. At a horizontal displacement of 10 mm (0.38% drift), the stiffness of W2 was 1.44 times that of W1. At a horizontal loading displacement between 20 mm and 30 mm, the stiffness of W2 was 1.3 times that of W1. The results indicated that the small grid size of the PP band mesh resulted in a higher stiffness of the hybrid structure than did the large grid size.

### 6.5. Energy Dissipation

**Figure 18:**
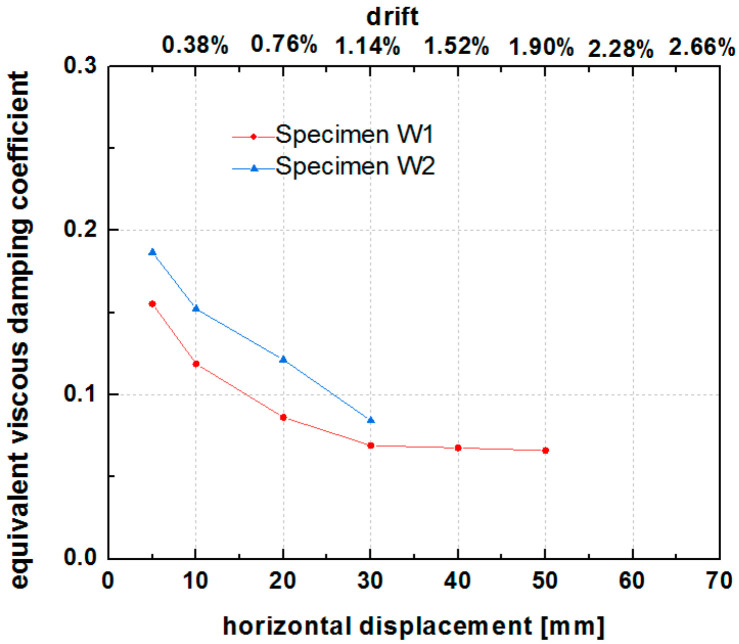
Equivalent viscous damping coefficient of specimens W1 and W2.

The equivalent viscous damping coefficient and cumulative energy are commonly calculated to access the energy dissipation capacity of a component or a structure. As shown in Figure 18, the equivalent viscous damping coefficient showed a decreasing trend in the range of 0.2 to 0.07 before 30 mm lateral displacement (1.14% drift), followed by stabilization at around 0.08. The grid size of the PP band mesh affected the equivalent viscous damping coefficient of the hybrid structure. The viscous damping coefficient was higher for W2 than W1 (0.08–0.2 for W2 and 0.07–0.16 for W1). An increase in the grid density of the PP band enhanced the equivalent viscous damping coefficient of the specimens.

**Figure 19:**
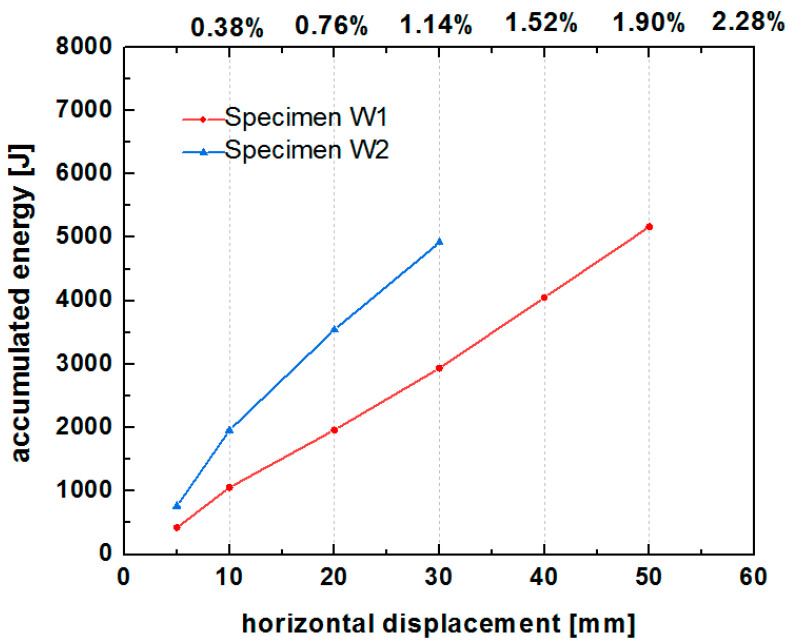
Cumulative energy of specimens W1 and W2.

The cumulative energy of the two specimens at different lateral displacements is shown in Figure 19. At a loading displacement from 0 to 30 mm, the cumulative energy was higher for specimen W2 (5000 J) than for specimen W1 (3000 J). In other words, the lateral drift of W2 was less than that of W1 (30 mm versus 50 mm) for dissipating the same amount of energy (5000 J). In addition, the cumulative energy increased as the grid density increased.

These results indicate that the narrow grid of the PP band mesh results in a higher energy dissipation capacity of the structure than the wide grid.

## 7. Comparison with an Unreinforced Specimen with Irregular Rubble Infill

We compared the experimental results with that of an unreinforced timber–stone hybrid wall with the same dimensions and irregular rubble without pebbles for the infill to assess the reinforcement effect. The experimental results of an unreinforced timber–stone hybrid wall subjected to a vertical load of 38 kN were reported in [62]. In this study, the unreinforced timber–stone hybrid wall is defined as reference specimen W0, which corresponds to specimen S2 in [62].

**Figure 20:**
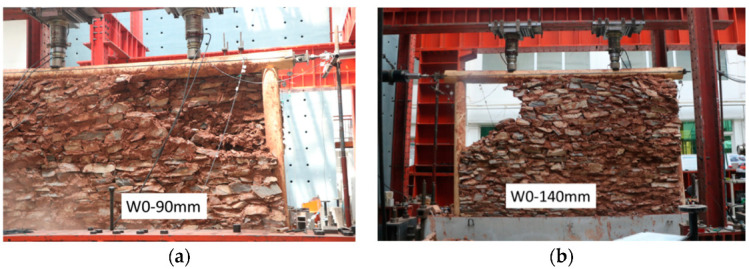
Displacement loading for specimen W0: (**a**) 90 mm and (**b**) 140 mm.

Unlike specimens W1 and W2, which exhibited stone collapses of large areas in the early loading phase, specimen W0 showed continuous falling of soil and stones due to dislocation during the long loading process. At a loading displacement of 90 mm, stones fell from a single layer at the wall corner (about one-sixth of the wall area; Figure 20a). The wall at the same position in the other layer bulged and cracked. At 140 mm horizontal displacement, a cavity occurred at one corner, representing about one-sixth of the wall area (Figure 20b). The other corner of the wall exhibited split layers with some stones about to fall.

**Figure 21:**
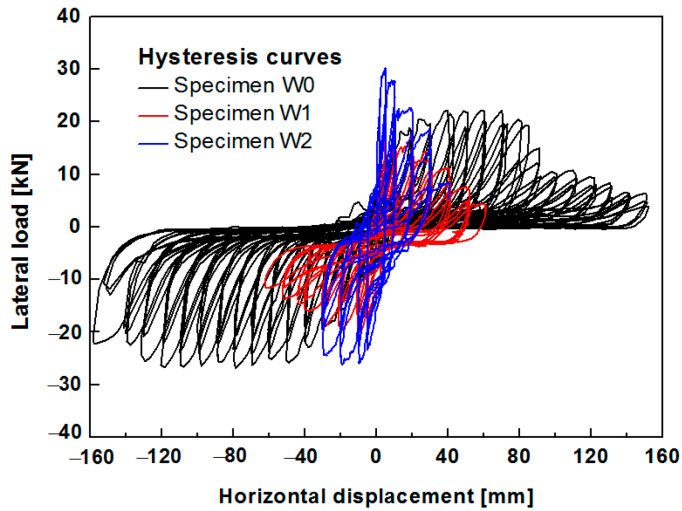
Hysteresis curves of specimens W0, W1, and W2.

The hysteresis loops of the unreinforced W0 and reinforced W1 and W2 specimens are depicted in Figure 21. The curve of specimen W0 exhibited plump ends and pinching in the middle, indicating high ductility. The main reason is the good bond between the stones and the clay, preventing stone dislocation and slip, unlike in the specimen with the pebbles. The hysteresis curve of the unreinforced W0 exhibits strong asymmetry because the wall collapsed at one corner during loading, whereas the remainder of the wall did not collapse due to good bonding between the stone blocks and the clay. Therefore, the peak compression between the wooden column and the wall at one side (negative direction) remained stable for a long time. The out-of-plane stone collapse behavior differed for the walls with and without pebbles. Although the PP band mesh was installed on one side of the wall, the wall with pebbles was more likely to collapse at the unreinforced side. Different levels of stone collapse occurred at both corners of the unreinforced single layer of the infill. The reinforced specimens W1 and W2 showed symmetrical behavior in the positive and negative hysteresis loops.

**Figure 22:**
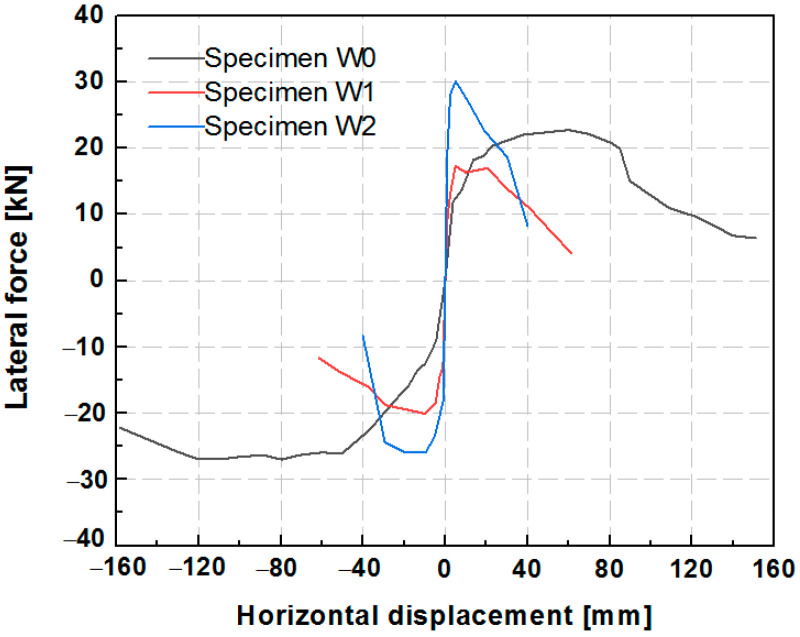
Hysteresis envelope curves of specimens W0, W1, and W2.

The hysteresis envelope curves of the three specimens are presented in Figure 22. Despite the one-sided reinforcement with the wide PP band mesh, specimen W1 exhibited a lower lateral force than the unreinforced specimen W0 without pebbles. It is possible that the unreinforced hybrid structure that included rubble and pebbles as infill provided a much lower lateral capacity due to stone dislocation, slip, or collapse. The narrow PP band mesh grid of specimen W2 enhanced the lateral strength, which was higher than that of the unreinforced specimen W0 and the reinforced specimen W1 with the wide PP band mesh grid. Table 4 lists the key seismic parameters, including the initial stiffness and strength, of the three specimens. The timber frame with rubble and pebble infill strengthened by the narrow PP band mesh grid (specimen W2) exhibited an average lateral resistance of 28.01 kN. This value was higher than that of specimen W0 (24.87 kN) and specimen W1 (18.65 kN). In addition, regardless of the type of stones in the infill, the reinforcement substantially improved the lateral stiffness of the hybrid structure, with average values of 10.61 kN/mm for W1, 18.98 kN/mm for W2, and 2.13 kN/mm for the unreinforced specimen W0. The reinforced hybrid walls had 5–9 times higher lateral stiffness than the unreinforced wall.

**Figure 23:**
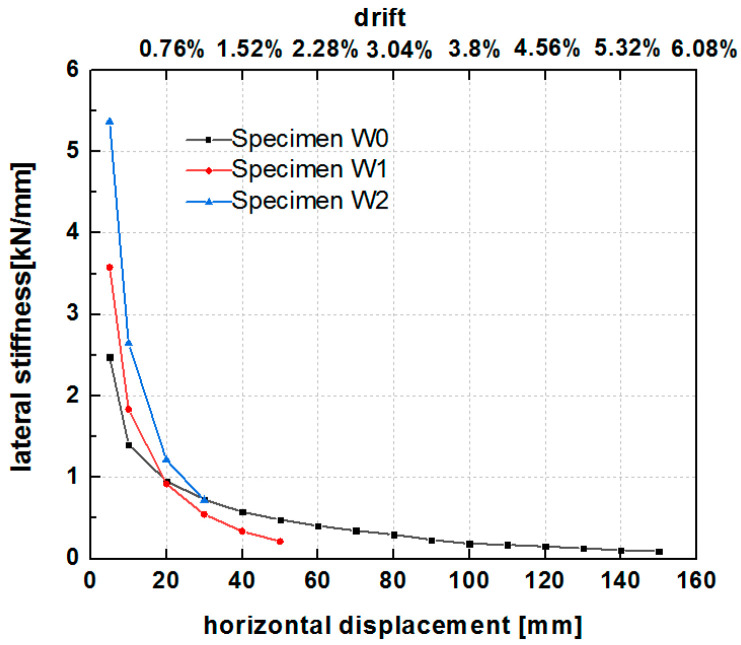
Stiffness degradation of specimens W0, W1, and W2.

The stiffness degradation of the unreinforced and reinforced specimens is presented in Figure 23. Although pebbles were used as part of the infill material of the reinforced specimens, the reinforced specimens had higher stiffness values (more than 1 kN/mm) than the unreinforced specimen without pebble infill before 20 mm horizontal loading displacement (0.76% drift). Subsequently, the degradation of the stiffness of the reinforced specimens was close to or lower than that of the unreinforced specimen due to the collapse of the pebbles in the early phase.

**Figure 24:**
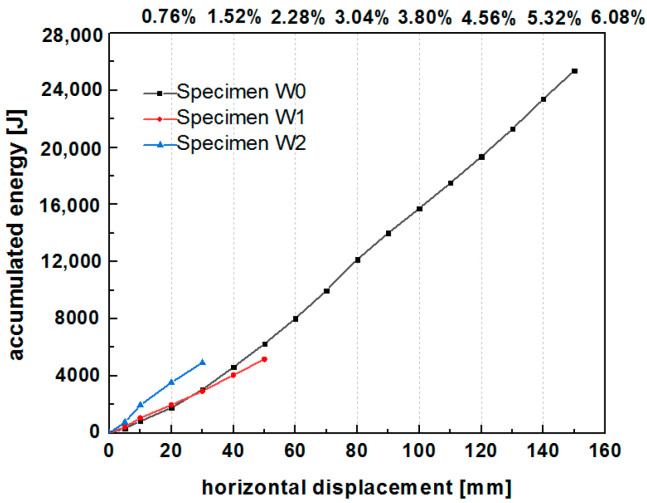
Cumulative dissipated energy of Specimens W0, W1, and W2.

The cumulative energy dissipation of the unreinforced and reinforced specimens is presented in Figure 24. It is clear that the cumulative energy dissipation curve of specimen W0 is much longer and higher than specimens W1 and W2, due to greater loading displacement up to 160 mm. The following discussion is mainly aimed to the same displacement range of three samples lower than 60 mm. The cumulative energy dissipation of specimen W1 strengthened by the wide PP band mesh grid was close to that of the unreinforced specimen W0 without pebble infill. For specimen W1, an increase in the energy dissipation capacity due to the reinforcement with the wide PP band mesh grid compensated for the lower energy dissipation caused by the presence of pebbles. The narrow PP band mesh grid significantly improves the energy dissipation capacity. Specimen W2 exhibited a cumulative energy dissipation of 5000 J, whereas that of W1 and W0 was about 3000 J at 30 mm load displacement.

## 8. Conclusions

This is the first study to use the PP band mesh retrofitting method for an existing timber frame with mud and stone infill structure. The PP band mesh was attached to the infill wall and was covered with plastering mortar. The PP band was also used to strengthen the connection between the infill and the timber frame by using steel strips and steel buckles. In-plane pseudo-static tests were conducted to investigate the effectiveness of the PP band mesh retrofitting method to improve the in-plane response of the hybrid timber frame–mud and stone infill structure and delay the collapse of the infill. Further research will be conducted on the out-of-plane behavior of PP band mesh retrofitting the structure and the results will be reported when available.

(1) Under low cyclic reversed loading, deformation incompatibility (alternate squeezing and detachment) occurred between the infill wall and the timber frame. The diagonal bracing effect and low bonding strength of the clay caused the crushing of the infill wall at the corner and out-of-plane stone failing. The PP band mesh retrofitting method considerably improved the structure’s seismic performance and minimized failure. The mortar covering the reinforcement mesh held the loose stones in place and caused the wall to act as an integrated structure. The PP band mesh connecting the infill and the timber frame not only prevented out-of-plane collapse at the corner of the reinforced wall due to a diagonal bracing effect, but also prevented the detachment of the reinforced side of the infill from the timber frame.

(2) The PP band mesh significantly improved the structure’s lateral performance. The hybrid wall with the narrow PP band mesh grid (150 mm × 150 mm) had a higher lateral stiffness (79%) and lateral capacity (50%) than the wall with the wide grid (250 mm × 250 mm). The PP band mesh considerably improved the energy dissipation capacity of the structure and changed the hysteresis curve shape from an 8-shape to a shuttle shape. However, although the narrow PP band mesh improved the stiffness and strength of the structure, it resulted in a lower ductility of the wall than the wide PP band mesh.

(3) Specimen W1, consisting of rubble and pebble infill, exhibited a lower lateral capacity than the unreinforced specimen W0 without pebble infill. It is possible that the lower lateral capacity was related to the lower integrity of the wall with the pebbles. However, specimen W2 with the narrow PP band mesh grid had a higher lateral capacity than both specimens W0 and W1. The reinforced hybrid walls had 5–9 times higher lateral stiffness than the unreinforced wall. Regardless of the type of stones in the infill, the reinforcement prevented stones falling from the reinforced side of the infill, which would protect the residents in the case of an earthquake.

(4) The improvement in the maximum lateral capacity dependent on the type of PP band mesh and cement mortar reinforcement on one side is limited. The compression of the timber frame resulted in the early detachment at the corners of the unreinforced side of the infill wall. We recommend the use of PP band mesh reinforcement on the inside of traditional timber–stone dwellings to preserve the buildings’ architectural style and features. The lateral stiffness of the timber frame should be strengthened by an angle brace to minimize the deformation incompatibility between the timber frame and infill, minimize the infill damage from the timber frame, and improve the lateral strength of the hybrid wall.

## Figures and Tables

**Table 1:** Specimen detail.

Specimen Label	Reinforcement Type	Infill Detail
W1	Strengthened by PP band mesh (250 mm × 250 mm) and plastering mortar	Pebbles and irregular stones infilled with mud
W2	Strengthened by PP band mesh (150 mm × 150 mm) and plastering mortar	

**Table 2:** Mechanical properties of the PP bands.

Sample Label	Size (mm)			Elastic Modulus (GPa)	Ultimate Tensile Force (kN)	Ultimate Tensile Strength (MPa)
	L_c_	a_0_	b_0_			
A3	100	1	13	1.9	1.60	123
A1	100	0.5	13.4	1	0.51	76
A2	100	0.8	11	0.5	0.51	58
A4	100	0.65	11.5	1.9	0.86	116
A5	100	0.75	11.4	1.9	1.25	146

Note: Lc—extensometer gauge length; a_0_—thickness of the PP band; b_0_—width of the PP band.

**Table 3:** Seismic parameters corresponding to W1 and W2.

		Initial State	Yield Limit State		Strength Limit State		Failure Limit State		Ductility
	Loading Direction	Ke	Pyield	Dyield	Ppeak	Dpeak	Pu	Du	*δ*
		[kN/mm]	[kN]	[mm]	[kN]	[mm]	[kN]	[mm]	
W1	Positive	9.06	15.96	1.76	17.24	5.14	13.79	30.42	17.2
	Negative	12.15	18.57	1.53	20.06	9.98	16.05	37.16	24.3
	Mean	10.61	17.27	1.645	18.65	7.56	14.92	33.79	20.75
W2	Positive	20.58	27.23	2.26	30.1	5.00	24.08	16.49	7.30
	Negative	17.37	24.59	2.37	25.92	9.52	20.74	31.78	13.4
	Mean	18.98	25.91	2.315	28.01	7.26	22.41	24.135	10.35

**Table 4:** The seismic parameters of the three specimens.

Label	Initial Stiffness [kN/mm]			Strength [kN]		
	Positive	Negative	Mean	Positive	Negative	Mean
W0	2.98	1.19	2.13	22.74	26.99	24.87
W1	9.06	12.15	10.61	17.24	20.06	18.65
W2	20.58	17.37	18.98	30.1	25.92	28.01

## Data Availability

The data in this paper are given in the tables and figures within the manuscript.

## References

[1] Xue J., Xu D., Ren G., Guo R. (2019). Shake table test on seismic performance of column and tie wooden structure in traditional residence. Chin. Civ. Eng. J..

[2] Wang H., Shang S., He F., Huang S., Deng T., Liu D. (2012). Shaking table tests of Chinese traditional wood building and light wood framed building. J. Build. Struct..

[3] Xue J., Xu D., Guo R. (2020). Shaking table tests and contrastive analysis for column-and-tie wooden buildings with and without infills. J. Vib. Shock.

[4] Xiong H., Wang J., Wu L., Chen L. (2018). Experimental study on lateral resistance performance of Chuandou wooden frame structures. J. Build. Struct..

[5] Makarios T., Demosthenous M. (2006). Seismic response of traditional buildings of Lefkas Island. Greece. Eng. Struct..

[6] Ruggieri N., Tampone G., Zinno R. (2015). Historical Earthquake-Resistant Timber Frames in the Mediterranean Area.

[7] Meireles H., Bento R., Cattari S., Lagomarsino S. (2012). A hysteretic model for “frontal” walls in Pombalino buildings. Bull. Earthq. Eng..

[8] Dutu A., Ferreira J., Guerreiro L., Branco F., Goncalves A.M. (2012). Timbered masonry for earthquake resistance in Europe. Mater. De Constr..

[9] Langenbach R. (2007). From “opus craticium” to the “Chicago frame”: Earthquake resistant traditional construction. Int. J. Archit. Herit..

[10] Poletti E., Vasconcelos G. (2015). Seismic behavior of traditional timber frame walls: Experimental results on unreinforced walls. Bull. Earthq. Eng..

[11] Yasemin D.A. (2017). Seismic resistance of traditional timber-frame hımış structures in Turkey: A brief overview. Int. Wood. Prod. J..

[12] Champagne F.V., Sieffert Y., Grange S., Polastri A., Daudeville L. (2014). Experimental analysis of seismic resistance of timber-framed structures with stones and earth infill. Eng. Struct..

[13] Dutu A., Niste A.M., Spatarelu A.I., Dima D.I., Kishiki S. (2018). Seismic evaluation of Romanian traditional buildings with timber frame and mud masonry infills by in-plane static cyclic tests. Eng. Struct..

[14] Cruz H., Pedro J., Stap M., Fronteira A.M.D., José L., Machado S., Lourenço P.B., Roca P. (2001). The Use of FRP in the Strengthening of Timber Reinforced Masonry Load-Bearing Walls.

[15] Vasconcelos G., Poletti E., Salavessa E., Jesus A.M., Loureno P.B., Pilaon P. (2013). In-plane shear behavior of traditional timber walls. Eng. Struct..

[16] Poletti E., Vasconcelo G. (2012). Seismic behavior of traditional half-timbered walls: Cyclic tests and strengthening solutions. J. Herit. Conserv..

[17] Ali Q., Schacher T., Ashraf M., Alam B., Naeem A., Ahmad M.N., Umar M. (2012). In-plane behavior of full scale Dhajji Walls (Wooden Braced with Stone Infill) under quasi static loading. Earthq. Spectra.

[18] Ehsani M.R. (1997). Strengthening of earthquake damaged masonry structures with composite materials. Non-Metallic (FRP) Reinforcement for Concrete Structure, Proceedings of the Second International Rilem Symposium, London, UK, October 1995.

[19] ElGawady M.A., Lestuzzi P., Badoux M. (2007). Static cyclic response of masonry walls retrofitted with fiber-reinforced polymers. J. Compos. Constr..

[20] Chagas J., Moita G. (2015). Influence of fibre reinforced polymers in the rehabilitation of damaged masonry wallettes. Appl. Adhes. Sci..

[21] Fam A., Musiker D., Kowalsky M., Rizkalla S. (2003). In-plane testing of damaged masonry wall repaired with FRP. Adv. Compos. Lett..

[22] Marcari G., Manfredi G., Pecce M. Experimental behavior of masonry panels strengthened with FRP sheets. Proceedings of the 6th Internaition Symposium on Fibre-Reinforced Polymer Reinforcement for Concrete Structures.

[23] Zhou D., Lei Z., Wang J. (2013). In-plane behavior of seismically damaged masonry walls repaired with external BFRP. Compos. Struct..

[24] Zhou D., Zhou S., Lei Z. (2016). In-Plane Shear Behaviors of Constrained Masonry Walls Externally Retrofitted with BFRP. J. Compos. Constr..

[25] Tumialan J.G., Morbin A., Micelli F., Nanni A. Flexural strengthening of URM walls with FRP laminates. Proceedings of the 3rd International Conference on Composites in Infrastructure.

[26] Yu W., Ge X., Zhu L. (2007). Experimental Study on Shaking Table Test of Adobe Building Model of Kashi, Xinjiang. Earthq. Resist. Eng. Retrofit..

[27] Jing D., Yan J., Cao S. (2016). Experimental Study on Seismic Performance of Brick Masonry Infill Walls with Wood Frame Strengthened by Embedded Steel Bars and External Steel Plates. J. Hunan Univ..

[28] Borri A., Corradi M., Castori G., Molinari A. (2019). Stainless steel strip–A proposed shear reinforcement for masonry wall panels. Constr. Build. Mater..

[29] Farooq S.H., Shahid I., Llyas M. (2014). Seismic performance of masonry strengthened with steel strips. KSCE J. Civ. Eng..

[30] Darbhanzi A., Marefat M.S., Khanmohammadi M. (2014). Investigation of in-plane seismic retrofit of unreinforced masonry walls by means of vertical steel ties. Constr. Build. Mater..

[31] Wight G.D., Ingham J.M., Wilton A.R. (2007). Innovative seismic design of a post-tensioned concrete masonry house. Can. J. Civ. Eng..

[32] Wight G.D., Kowalsky M.J., Ingham J.M. (2007). Shake table testing of posttensioned concrete masonry walls with openings. J. Struct. Eng..

[33] Renle M.A., Lu J.A., Minjuan H.A., Cheng F.B., Feng L.C. (2012). Experimental investigations on masonry structures using external prestressing techniques for improving seismic performance. Eng. Struct..

[34] Yang K.H., Joo D.B., Sim J.I., Kang J.H. (2012). In-plane seismic performance of unreinforced masonry walls strengthened with unbonded prestressed wire rope units. Eng. Struct..

[35] Liu H., Yue Y., Han M., Tian Y. (2020). Application of prestressed seismic retrofitting technology in strengthening and reconstruction of rural dilapidated residences. Build. Struct..

[36] Liu H., Han M., Lan C., Wang T., Tian Y. (2016). Pseudo-dynamic test and quasi-static test of full-scale model of a two-story brick building retrofitted with prestressed tendons. Chin. Civ. Eng. J..

[37] Papanicolaou C.G., Triantafillou T.C., Karlos K., Papathanasiou M. (2007). Textile reinforced mortar (TRM) versus FRP as strengthening material of URM walls: Out-of-plane cyclic loading. Mater. Struct..

[38] Arisoy B., Ercan E., Demir A. (2015). Strengthening of brick masonry with PVA fiber reinforced cement stucco. Constr. Build. Mater..

[39] Lin Y.W., Wotherspoon L., Scott A., Ingham J.M. (2014). In-plane strengthening of clay brick unreinforced masonry wallettes using ECC shotcrete. Eng. Struct..

[40] Deng M., Dong Z., Yang S., Wang L., Zhou T. (2019). Shaking table test on damaged masonry structure reinforced with high ductile concrete layer. Eng. Mech..

[41] Zhou T., Liu B., Shi Q., Zhang B. (2019). Seismic retrofitted experiment on the timber frame-cavity wall houses by shaking table test. World Earthq. Eng..

[42] Kadam S.B., Singh Y., Bing L. (2014). Strengthening of unreinforced masonry using welded wire mesh and micro-concrete—Behaviour under in-plane action. Constr. Build. Mater..

[43] Shermi C., Dubey R.N. (2018). In-plane behaviour of unreinforced masonry panel strengthened with welded wire mesh and mortar. Constr. Build. Mater..

[44] Messali F., Meteili G., Plizzari G.A. (2017). Experimental results on the retrofitting of hollow brick masonry walls with reinforced high performance mortar coatings. Constr. Build. Mater..

[45] Zhao Z., Xu S., Chen Y., Liu H. (2018). Seismic behavior of a retrofitted one-story four-bay masonry residential building in rural area of Beijing. J. Build. Struct..

[46] Kadam S.B., Singh Y., Li B. (2015). Out-of-plane behaviour of unreinforced masonry strengthened using ferrocement overlay. Mater. Struct..

[47] Navaratnarajah S., Meguro K. Seismic retrofitting of non-engineering masonry houses using polypropylene band meshes. Proceedings of the 2nd International Symposium on Advances in Civil and Environmental Engineering.

[48] Mayorca P., Meguro K. Efficiency of polypropylene bands for the strengthening of masonry structures in developing countries. Proceedings of the 5th International Summer Symposium.

[49] Mayorca P., Meguro K. Proposal of an Efficient Technique for Retrofitting Unreinforced Masonry Dwellings. Proceedings of the 13th World Conference on Earthquake Engineering.

[50] Sugijopranoto A., Triwiyono A., Priyosulistyo H. (2017). Experimental Investigation on Pre-stressed Polypropylene-band. Procedia Eng..

[51] Sathiparan N., Mayorca P., Neshali K.N., Guragain R., Meguro K. (2006). Experimental study on unburned brick masonry wallettes retrofitted by PP-band meshes. Seisan Kenkyu.

[52] Sathiparan N., Mayorca P., Meguro K. Parametric study on diagonal shear behavior of masonry wall retrofitted by PPband mesh. Proceedings of the 26th Annual Conference of Japan Society for Natural Disaster Science.

[53] Sathiparan N., Mayorca P., Meguro K. (2012). Shake Table Tests on One-Quarter Scale Models of Masonry Houses Retrofitted with PP-Band Mesh. Earthq. Spectra.

[54] Sathiparan N. (2018). Effect of Roof and Diaphragm Connectivity on Dynamic Behaviour of the PP-band Retrofitted Adobe Masonry Structures. Period. Polytech. Civ. Eng..

[55] Sathiparan N., Meguro K. (2015). Strengthening of adobe houses with arch roofs using tie-bars and polypropylene band mesh. Constr. Build. Mater..

[56] Zhou Q., Yang L., Zhao W. (2020). Experimental Analysis of Seismic Performance of Masonry Shear Wall Reinforced with PP-Band Mesh and Plastering Mortar under In-Plane Cyclic Loading. Adv. Civ. Eng..

[57] Banerjee S., Nayak S., Das S. (2019). Enhancing the flexural behaviour of masonry wallet using PP band and steel wire mesh. Constr. Build. Mater..

[58] (2009). Standard for Test Method of Performance on Building Mortar.

[59] (2018). Plastics-Determination of Tensile Properties.

[60] (2011). Standard Test Methods for Cyclic (Reversed) Load Test for Shear Resistance of Walls for Buildings.

[61] (2015). Specification for Seismic Test of Buildings.

[62] Shen Y., Yan X., Yu P., Liu H., Wu G., He W. (2021). Seismic Resistance of Timber Frames with Mud and Stone Infill Walls in a Chinese Traditional Village Dwelling. Buildings.

